# Involvement of hedgehog pathway in early onset, aggressive molecular subtypes and metastatic potential of breast cancer

**DOI:** 10.1186/s12964-017-0213-y

**Published:** 2018-01-08

**Authors:** Syeda Kiran Riaz, Jahangir Sarwar Khan, Syed Tahir Abbas Shah, Fen Wang, Lin Ye, Wen G. Jiang, Muhammad Faraz Arshad Malik

**Affiliations:** 1 Department of Biosciences, COMSATS Institute of Information Technology, Park Road, Islamabad, Zip code: 44000 Pakistan; 2 Department of Surgery, Holy Family Hospital, Rawalpindi Medical University, Rawalpindi, Pakistan; 3 Center for Cancer and Stem Cell Biology, Institute of Biosciences and Technology, Texas A&M Health Science Center, Houston, USA; 4 Cardiff China Medical Research Collaborative, School of Medicine, Cardiff University, Cardiff, UK

**Keywords:** Breast cancer, Hedgehog pathway, SHH, DHH, GLI1, GANT61

## Abstract

**Background:**

Dysregulation of hedgehog pathway is observed in numerous cancers. Relevance of hedgehog pathway genes in cancer cohort and inhibition of its downstream effector (GLI1) towards metastasis in cell lines are explored in the study.

**Method:**

One hundred fifty fresh tumours of breast cancer patients were collected for the study. Based on differential expression, panel of 6 key regulators of the pathway (SHH, DHH, IHH, PTCH1, SMO and GLI1) in microarray datasets were identified. Expressional profiles of aforementioned genes were later correlated with clinico-pathological parameters in Pakistani breast cancer cohort at transcript and protein levels. In addition, GLI1 over expressing breast cancer cell lines (MDA-MB-231 and MCF-7) were treated with GANT61 to explore its probable effects on metastasis.

**Result:**

SHH, DHH, PTCH1 and GLI1 were significantly over-expressed in tumours as compared with respective normal mammary tissues. A significant correlation of SHH, DHH and GLI1 expression with advanced tumour size, stages, grades, nodal involvement and distant metastasis was observed (*p* < 0.05). Over-expression of SHH, DHH and GLI1 was significantly related with patients having early onset and pre-menopausal status. Of note, hedgehog pathway was frequently up regulated in luminal B and triple negative breast cancer affected women. In addition, positive correlations were observed among aforementioned members of pathway and Ki67 (r-value: 0.63–0.78) emphasizing their role towards disease progression. Exposure of GANT61 (inhibitor for GLI1) significantly restricted cell proliferation, reduced cell motility and invasion.

**Conclusion:**

Role of activated hedgehog pathway in breast cancer metastasis provides a novel target for cancer therapy against aggressive cancer subtypes.

**Electronic supplementary material:**

The online version of this article (10.1186/s12964-017-0213-y) contains supplementary material, which is available to authorized users.

## Background

Breast cancer is among the most common malignancies accounting for 25% of total cancer burden and 15% of cancer related deaths [1]. Recently, reactivation of early developmental pathways in promoting solid tumour growth has been observed [2]. Pivotal role of hedgehog pathway in embryonic patterning and later mammary gland development has been reported [3]. Briefly, binding of three ligands Sonic hedgehog (SHH), Indian hedgehog (IHH) and Desert hedgehog (DHH) with Patched1 (PTCH1) results in release of Smoothened (SMO). Once relieved SMO activates GLI (glioma-associated oncogene homolog 1, transcription factor) responsible for activation of several downstream growth effectors [3].

Numerous studies suggested dysregulation of hedgehog pathway has an important impact on carcinogenesis [4–6]. Exploring any probable correlation of hedgehog pathway with clinico-pathological parameters is a step forward towards development of prognostic and predictive markers for future therapeutics. Earlier, SHH and GLI1 over-expression have been associated with poor prognosis in different types of cancers including breast [6, 7], colon [8], glioma [9] and prostate [10]. However, prognostic significance of hedgehog pathway with early disease onset and different molecular subtypes of breast cancer remain contradictory. Furthermore, its contribution towards metastasis by using cancer cell lines is yet to be established. Aim of the study was initially to observe hedgehog pathway activation both at mRNA and protein levels in the cohort. Based on these findings, significance of hedgehog pathway in breast cancer metastasis was explored using GANT61 (GLI antagonist). Blockade of GLI1 via GANT61 will be helpful to provide novel insight for involvement of hedgehog pathway in disease progression.

## Methods

### Publicly available expression data assessment

Pre-evaluation of hedgehog pathway in breast cancer datasets of two publicly available repositories namely, Oncomine and cBioportal were performed. Oncomine (Compendia Bioscience, Ann Arbor, MI; http://www.oncomine.org/) is an online database consisting of earlier published 715 microarray datasets. RNA expression status of SHH, DHH, IHH, PTCH1, SMO and GLI1 genes were evaluated in 593 TCGA breast cancer samples and 59 Finak breast cancer samples available in Oncomine database. In order to score gene as positive (high expression) or negative (low expression), the Log2 Median-Centered ratio, as reported in the Oncomine database, was used to evaluate differential expression of these genes in different clinical groups.

Similarly, cBioportal for Cancer Genomics (http://www.cbioportal.org) an online publicly available database, was screened in extended TCGA dataset of 1105 breast cancer patients [11]. RNAseq data of TCGA cohort was explored to validate expression pattern of SHH, DHH, IHH, PTCH1, SMO and GLI1.

### Clinical characteristics of the cohort

Tumour biopsies (*n* = 150) along with adjacent normal mammary tissues were collected immediately after surgery and snap frozen till further usage. Data regarding clinical and pathological findings were obtained in subsequent follow-up from respective laboratory reports and consultation.

### RNA isolation and cDNA synthesis

RNA isolation was performed with TRIzol® (15596–018, Invitrogen, USA) from the respective biological specimens following manufacturer’s instructions. Briefly, 1cm^3^ tissue section was homogenized using a hand held homogenizer in ice-cold RNA extraction buffer. Concentration of RNA was determined using nanodrop (Nanophotometer®Pearl, Implen, Germany). cDNA was synthesized using Revert Aid First Strand cDNA Synthesis Kit (K1622, Thermo Scientific, USA).

### Quantitative real-time PCR

Primers for hedgehog pathway genes (SHH, DHH, IHH, PTCH1, SMO and GLI-1) were designed and synthesized from Integrated DNA Technology. β-actin was used to normalize the data. Sequences along with their amplicon sizes are provided in the Additional file 1: Table S1. Furthermore, primers for biomarkers ER (Estrogen receptor), PR (Progesterone receptor), HER-2 (Human Epidermal growth factor Receptor 2) and Ki-67 (Proliferative marker) used for molecular subtyping of breast cancer were also synthesized from IDT. VeriQuest SYBR Green qPCR Master Mix (75600200RXN, Thermo Scientific, USA) was used for qPCR in *Step One plus* (Applied Biosystem, USA). Reaction conditions included an initial denaturation at 95 °C for 15 min, followed by 40 cycles of denaturation at 95 °C for 15 s and annealing at 55 °C for 1 min in each cycle. Relative mRNA expression was calculated using the 2^−ΔΔCt^ method. QCanvas was used for the hierarchical clustering and visualizing heatmap of SHH, DHH, IHH, PTCH and SMO based on relative expression values [12].

### Immunohistochemistry

Immunohistochemical staining was done using both tumour and normal frozen sections of 4 μm thickness of given cohort bio-specimens. Initially, these slices were mounted on Super Frost Plus microscopic slides and air-dried for 30 min. These fixed tissue sections were treated with 50% methanol and 50% acetone for 15 min. Sections were then air dried for 10 min and stored at −20 °C (wrapped in foil) till further usage. Briefly, these slides were placed in PBS for 5 min to rehydrate, followed by blocking with 10% horse serum. These sections were later exposed with primary antibodies for SHH, DHH and GLI1. Antibodies used were rabbit anti-SHH polyclonal antibody (H-160; sc-9024, dilution 1:200; Santa Cruz Biotechnology, USA), mouse monoclonal anti-DHH (sc-271,168, dilution 1:200; Santa Cruz Biotechnology, USA) and mouse anti-GLI1 monoclonal antibody (D1; sc-271,075, diluted 1:200; Santa Cruz Biotechnology, USA). Exclusion of primary antibody acted as a negative control. Immunostaining was performed as previously described [13]. Immuno-Reactive-Scores (IRS) were evaluated as the product of % of cells positively stained for each molecule categorized from 1 to 4 (1 = <25%, 2 = 25–50%, 3 > 50%). Final IRS scores were ranked as high or low based on mean of IRS. Expression of pathway molecules was correlated with demographic and clinico-pathological findings of the cohort.

### Breast cancer cell lines maintenance and culture condition

Breast cancer cell lines (MCF-7 and MDA-MB-231) were generously provided by Dr. Yi Li (Breast centre Li, Baylor College of Medicine, USA). These lines were cultured and maintained as recommended by ATCC. Role of hedgehog pathway towards metastasis was assessed in these cell lines by inhibiting downstream effector GLI via GANT61 (G9048-5MG, Sigma, Germany).

### Western blot analysis

Cells were homogenized in lysis buffer containing protease inhibitor. Concentration of the harvested proteins was determined using the Pierce BCA (Bicinchoninic Acid) protein assay kit (23,225, Thermo Scientific, USA). Western blotting was performed following protocol described previously [14]. Both primary and secondary antibodies used for this study were purchased from Santa Cruz Biotechnology (USA) unless stated otherwise. Human SHH (H-160; sc-9024, dilution 1:1000), human PTCH-1 (H-267; sc-9016, dilution1:500), human GLI-1 (H-300; sc-20,867, dilution 1:1000) were used. Rabbit polyclonal antibody was used as secondary antibody for these blots. Mouse monoclonal antibody targeting against human Actin (C-4; sc-47,778, dilution 1:3000) was used as control. The experiments were repeated three times independently.

### Cell proliferation assay

The cell proliferation assay was assessed with CCK-8 (CK04–05, Dojindo, Japan) as reported [15]. Briefly, triplicate sets of 96 well plates retaining 5x10^3^cells from both MDA-MB-231 and MCF-7 cell lines were prepared. After exposure with variable concentration (0, 5 μM, 10 μM, 15 μM and 20 μM) of GANT61, these plates were incubated at 37 °C retaining 5% CO_2_ for 48 h. Assay was repeated at least three times to validate IC50 value for GANT61. Furthermore, time dependent effect of GANT61 was evaluated at different time points (0, 24, 48, 72 and 96 h).

### Apoptosis assay

Briefly 1 × 10^5^ cells per well were cultured in 12 well plate, treated with variable concentrations of GANT61 (0, 5, 10, 15, 20 μM) for 48 h. Cells were collected after trypsinization and suspended in 1X binding buffer. Apoptotic cells were then stained by Annexin V-Cy3 (K102–100, BioVision, USA) and analyzed by flow cytometry as per manufacturer’s instructions.

### Cell invasion assay

Using inserts of 8 μm size placed in 24′ well plate, invasion assays were performed. Briefly, each insert was initially coated with Matrigel (356,234, BD Matrigel™ Basement Membrane Matrix, BD Biosciences, UK). After rehydration, 5 × 10^4^ cells of respective cell lines were seeded separately in the insert. Two set of inserts were used for each cell line. Cells in one insert were exposed to DMSO (Dimethyl sulfoxide) as control while in other insert GANT61 (10 μM) was added. After 24 h of incubation, inserts were fixed with formalin and stained in crystal violet*.* Cells were counted under microscope to observe any possible invasion as mentioned [14].

### Cell migration assay

A wound of ~300 μm was artificially introduced into monolayer of breast cancer cell line and cells movement towards the wound was measured over span of 48 h. Effect of GANT61 exposure towards cells motility was also calculated in these cell lines. Images were taken through a phase-contrast microscope (10× objective lens) at 0, 12, 24, 36 and 48 h. The wound area was quantified with the Image J software [16]. Data was expressed as the mean of three independent experiments ± standard deviation.

### Statistical analysis

Wilcoxon signed rank test was used to evaluate difference between tumours and adjacent controls. Mann Whitney and Kruskal-Wallis Anova were applied to assess the association of molecules with different clinico–pathological parameters. Spearman correlation was also used to evaluate relationship between all molecules in tumourigenesis. Unpaired t-test was applied to assess statistical differences in cell proliferation, migration and invasion assays. Kaplan-Meier plots were generated and survival was compared using log rank test. Cox’s proportional hazard regression analyses were performed to evaluate hazard ratio of covariates for overall survival of patients. Statistical analyses were performed using Graphpad Prism 5 (GraphPad Software, Inc. CA, USA) and StatsDirect (http://www.statsdirect.com. StatsDirect Ltd. 2013, England). The value for *p* < 0.05 was considered significant.

## Results

### Clinical and molecular characterization of cohort

In Oncomine, 2 datasets TCGA Breast and Finak Breast were assessed for expression variation of hedgehog pathway molecules in deposited data. SHH was significantly over-expressed in TCGA (F.C = 1.5, *p* < 0.05) and Finak dataset (F.C = 1.2, *p* < 0.0001) as compared to normal tissues. Expression of PTCH1 (F.C = 2.3, *p* < 0.0001) and GLI1 (F.C = 2.2, *p* < 0.0001) was also significantly elevated in Finak dataset (Additional file 1: Figure S1). Elevated expression of DHH, PTCH1, and GLI1 in the data deposited for TCGA provisional dataset in cBioPortal was also observed.

Demographic data of present study cohort comprised of 52% of patients having early disease onset and were ≤45 yrs. of age at the time of diagnosis. Around 60% of cohort represented moderately differentiated tumours. Two third of total cases were in advance stages of breast carcinogenesis. Distribution of these details along with clinical findings and assessment of hedgehog pathway are presented in the Table 1. For transcriptional analysis, fold change was evaluated relative to normal control tissues whose mean was calculated as 1.

**Table 1:** Clinical correlation of hedgehog pathway with demographic and pathological variables in Pakistani breast cancer cohort

Variable	Total	Mean ± SD											
		SHH	*p*-value	DHH	p-value	IHH	p-value	PTCH	p-value	SMO	p-value	GLI-1	p-value
Tumour	150	4.4 ± 3.3	< 0.0001	4.7 ± 3.5	< 0.0001	0.04 ± 0.2	< 0.0001	3.4 ± 3.1	< 0.0001	1.4 ± 2.1	0.61	3.8 ± 2.5	< 0.0001
Control	150	1.0 ± 0.9		1.0 ± 1.4		1.0 ± 1.1		1.0 ± 1.0		1.0 ± 1.3		1.0 ± 0.8	
Disease onset age													
Age ≤ 45 years	76	5.6 ± 3.9	0.03	5.5 ± 4.2	0.04	0.2 ± 0.8	0.65	3.2 ± 3.1	0.26	1.9 ± 2.2	0.03	6.3 ± 4.5	0.03
Age ≥ 45 years	74	4.2 ± 3.3		3.9 ± 2.9		0.04 ± 0.2		3.6 ± 3.0		1.2 ± 1.8		4.2 ± 2.7	
Menopausal status													
Pre-Menopausal	80	6.8 ± 4.8	0.0065	5.6 ± 3.9	0.01	0.23 ± 0.8	0.45	0.2 ± 0.8	< 0.0001	2.8 ± 3.6	0.12	7.9 ± 4.3	< 0.0001
Post-Menopausal	70	4.5 ± 3.5		3.9 ± 3.0		0.05 ± 0.2		3.9 ± 3.3		2.1 ± 3.1		3.9 ± 2.5	
Laterality													
Right	75	4.2 ± 2.8	0.01	5.8 ± 4.0	0.001	0.2 ± 0.8	0.13	3.4 ± 2.9	0.97	2.5 ± 2.9	0.26	4.5 ± 2.9	0.02
Left	75	6.1 ± 4.1		3.8 ± 3.0		0.1 ± 0.4		3.4 ± 3.2		2.1 ± 3.1		6.4 ± 4.5	
Grade-wise distribution													
Grade I	14	3.5 ± 2.7	0.0001	3.4 ± 2.4	0.48	0.2 ± 0.9	0.2	2.9 ± 2.9	0.68	2.4 ± 3.5	0.11	2.8 ± 1.9	0.01
Grade II	90	5.0 ± 3.8		4.9 ± 3.7		0.2 ± 0.6		3.4 ± 3.2		2.6 ± 3.2		3.2 ± 1.8	
Grade III	46	9.4 ± 6.5		4.9 ± 3.6		0.09 ± 0.3		3.6 ± 3.2		2.1 ± 3.6		5.6 ± 4.3	
Stage-wise distribution													
Stage I	34	4.2 ± 3.3	< 0.0001	6.2 ± 3.6	< 0.0001	0.1 ± 0.5	0.03	3 ± 2.8	0.35	2.6 ± 3.3	0.09	6.7 ± 3.9	0.005
Stage II	68	7.1 ± 4.1		8.3 ± 3.7		0.2 ± 0.7		3.3 ± 3.2		2.3 ± 2.7		7.2 ± 3.6	
Stage III	43	8.7 ± 3.9		8.6 ± 3.8		0.1 ± 0.2		3.6 ± 2.9		1.3 ± 1.7		7.9 ± 3.8	
Stage IV	5	14.6 ± 2.9		17 ± 2.2		0.06 ± 0.1		4.9 ± 2.8		2.3 ± 1.9		14.5 ± 3	
Nodal Involvement													
N0 (none)	48	4.3 ± 3.2	< 0.0001	3.3 ± 2.8	0.01	0.2 ± 0.5	0.02	2.9 ± 3	0.34	2.6 ± 3.2	0.92	4.1 ± 2.7	0.003
N1(lymph node < 4)	78	6.1 ± 4.1		5.8 ± 4.2		0.2 ± 0.7		3.6 ± 3.2		2.3 ± 3.3		5.9 ± 3.6	
N2(lymph node > 4)	24	8.2 ± 3.7		6.5 ± 4.4		0.006 ± 0		3.6 ± 2.8		2.7 ± 3.9		6.6 ± 3.9	
Distant Metastasis													
M0	145	4.4 ± 3.4	0.0003	4.5 ± 3.4	0.0002	0.14 ± 0.6	0.09	3.3 ± 3.0	0.12	2.4 ± 3.4	0.34	3.8 ± 2.5	0.0002
M1	5	14.6 ± 2.9		17 ± 2.2		0.06 ± 0.1		4.9 ± 2.8		2.3 ± 1.8		14.5 ± 3	
Molecular subtypes													
HER-2	21	2.7 ± 2.4	< 0.0001	2.2 ± 1.1	< 0.0001	0.03 ± 0.1	0.73	4.3 ± 4.5	0.04	2.5 ± 3	0.63	3.7 ± 2.1	0.0007
Luminal A	23	5.3 ± 2.8		1.9 ± 1.6		0.06 ± 0.2		2.1 ± 1.9		1.9 ± 2.7		2.7 ± 1.6	
Luminal B	79	6.7 ± 2.7		4.9 ± 3.5		0.1 ± 0.4		3.7 ± 3		2.6 ± 3.4		5.2 ± 2.9	
Triple Negative	27	5.9 ± 3.2		2.9 ± 2.6		0.3 ± 0.8		4.1 ± 3.8		1.8 ± 2.8		3.8 ± 2.5	

**Fig. 1:**
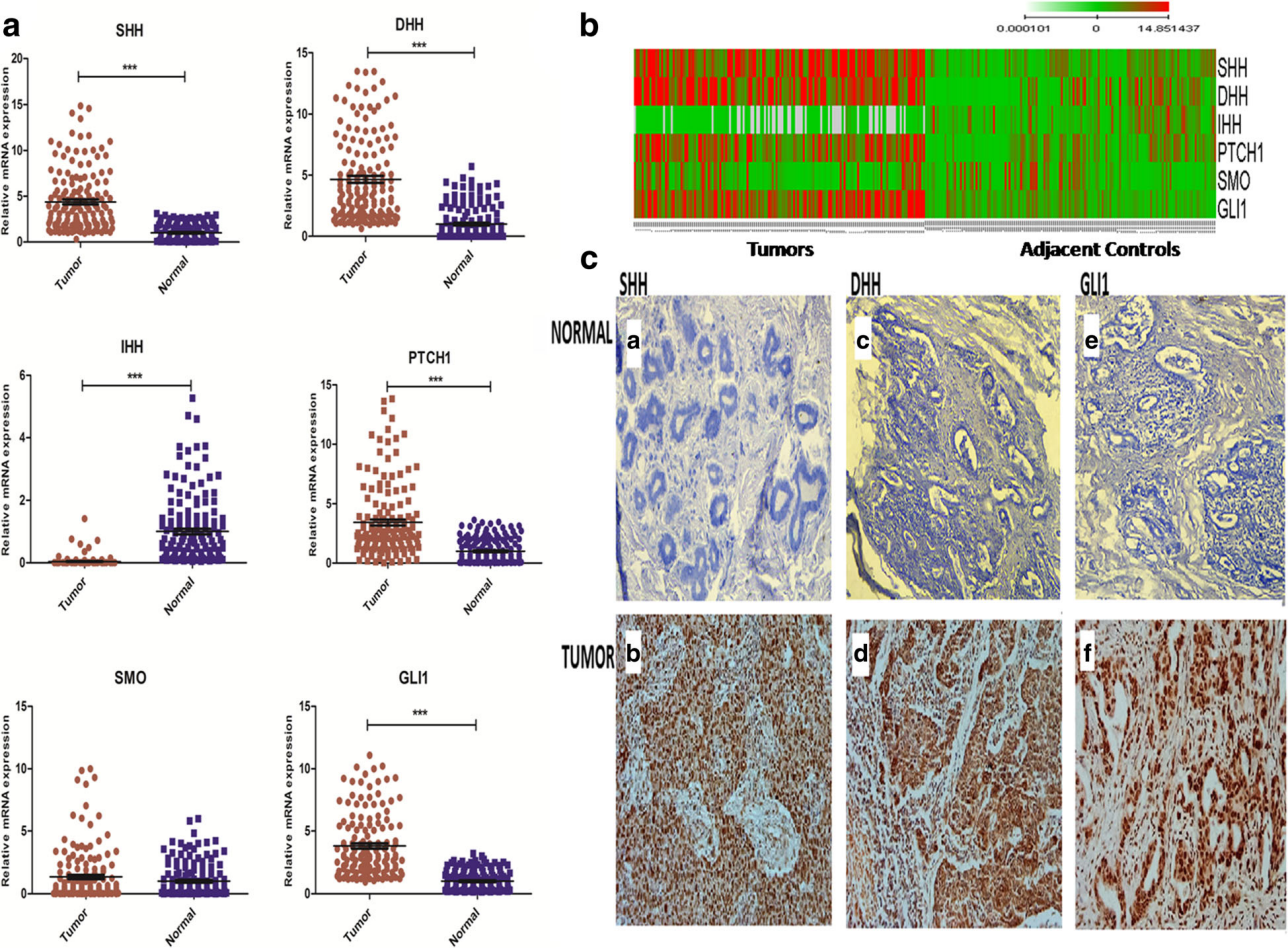
Expression Profiling of Hedgehog Pathway Members in Pakistani Cohort. **a**. Scatter plots showing mean relative mRNA expression of SHH, DHH, IHH, PTCH1, SMO and GLI1 in tumor samples as compared to normal tissues. **b**. Heatmap showing differential expression of analyzed molecules in tumors on left side and adjacent normal mammary tissues on right side. **c**. Immunostaining of SHH (A, B), DHH (C, D) and GLI1 (E, F) in normal tissues (A, C, E) and tumor tissues (B, D, F). (Wilcoxon Signed Rank Test, *p* < 0.0001)

Significant over expression of SHH (R.E = 4.4 ± 3.32), DHH (R.E = 4.7 ± 3.46), PTCH1 (R.E = 3.4 ± 3.08) and GLI1 (R.E = 3.8 ± 2.49) were detected in the breast tumour biopsies as compared with controls. In contrast, IHH (R.E = 0.04 ± 0.16) expression was significantly reduced in cancerous tissues. Lack of any significant association of SMO expression among tumour and controls were observed. PTCH1, SHH, DHH, and GLI1 over-expression were observed in 74%, 95%, 92% and 98% tumour specimens of the cohort. Expression patterns of these genes at mRNA and protein levels along with heatmap of qPCR data are shown in Fig. 1.

### Correlation of hedgehog pathway with patients having early disease onset and pre-menopausal status

**Fig. 2:**
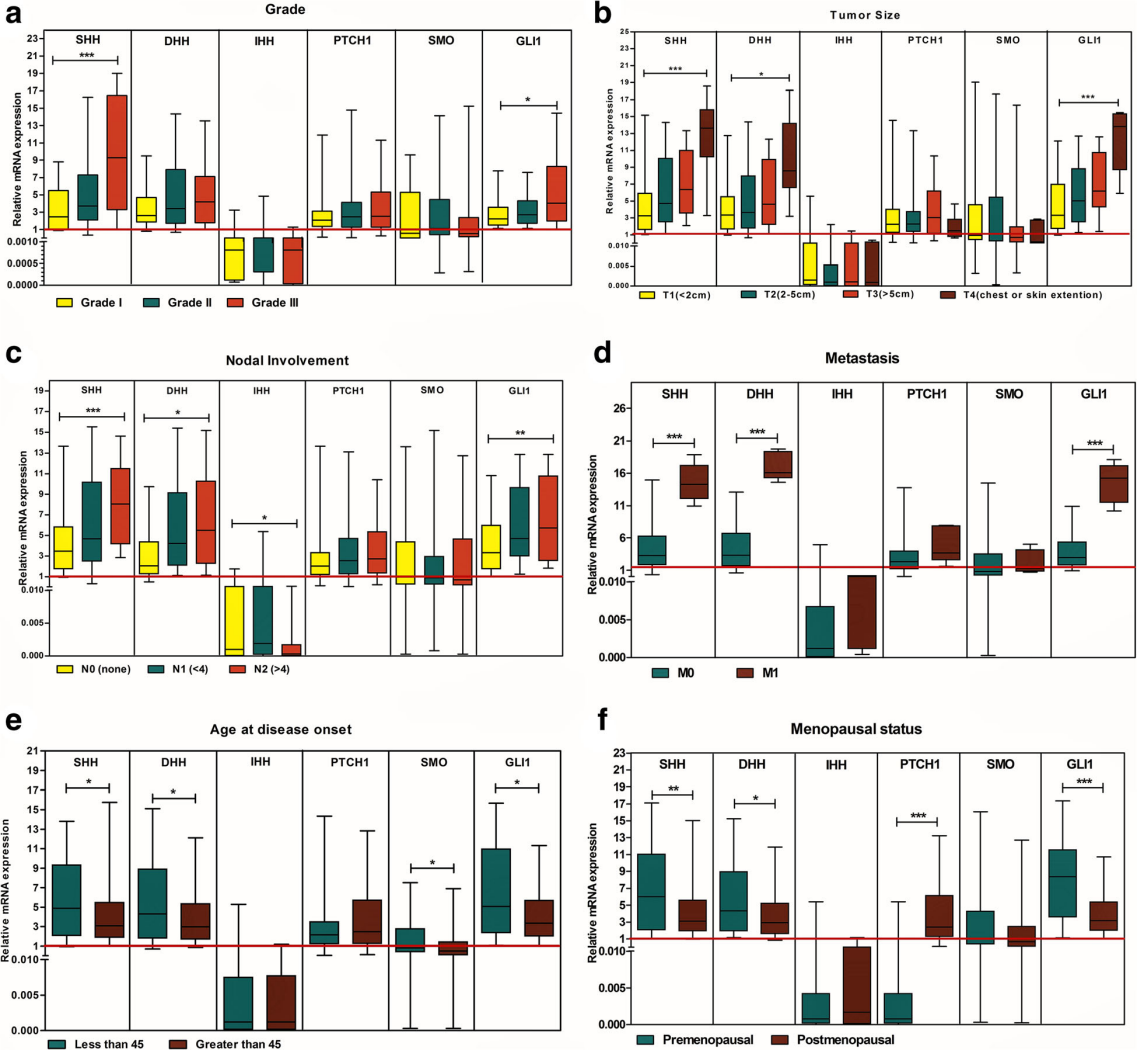
Transcript Profiling of Hedgehog Pathway using Box Whisker Plots. Association of relative expression of hedgehog pathway components with **a**. Nuclear grades, **b**. Tumor size, **c**. Nodal involvement, **d**. Metastasis, **e**. Age at disease onset, **f**. Menopausal status. (Kruskal-Wallis Anova (**a**, **b** and **c**) and Mann-Whitney U test (**d**, **e** and **f**), *p < 0.05, **p < 0.001, ***p < 0.0001)

In both datasets of Oncomine (TCGA breast and Finak breast) expression of SHH, DHH and GLI1 was higher in patients having early disease onset. Expression of SHH and GLI1 was also elevated in patients having pre-menopausal status while DHH was higher in post-menopausal cases. In the current study, cohort elevated expression of SHH (R.E = 5.6 ± 3.9, *p* < 0.05), DHH (R.E = 5.5 ± 4.1, *p* < 0.05), SMO (R.E = 1.9 ± 2.2, *p* < 0.05) and GLI1 (R.E = 6.3 ± 4.5, *p* < 0.05) correlated significantly with patients having early disease onset. Similarly, expression of SHH (R.E = 6.8 ± 4.8, *p* < 0.05), DHH (R.E = 5.5 ± 3.9, *p* < 0.05) and GLI1(R.E = 7.9 ± 4.4, *p* < 0.01) was significantly elevated in pre-menopausal patients while PTCH1(R.E = 3.9 ± 3.3, p < 0.01) was higher in post-menopausal (Fig. 2).

### Correlation of hedgehog pathway with markers of poor prognosis

Over expression of SHH, DHH and GLI1 were associated with cell grades in Finak dataset and also related with poorly differentiated tumours. Similarly, transcript copy numbers of SHH and DHH were also found related with advanced stages (III, IV). Elevated transcriptional profiles of SHH, DHH and GLI1 were associated with almost all indicators of poor prognosis in this cohort. Expression of SHH (*p* < 0.05) was 9 folds higher and GLI1 (*p* < 0.05) was 4 folds higher in patients presented with poorly differentiated histological grade. Mean mRNA values of SHH, DHH and GLI1 showed gradual increase with tumour size, nodal spread and distant metastasis. Significant correlation was observed between SHH (R.E = 12.76 ± 5.12, *p* < 0.0001), DHH (R.E = 9.8 ± 5.1, *p* < 0.05) and GLI1 (R.E = 12.3 ± 3.9, *p* < 0.0001) and invasive tumour size (Fig. 2). Expression variation of hedgehog pathway and its association with clinico-pathological parameters is illustrated in Table 1. SHH, DHH and GLI1 were also evaluated at protein level using IHC. Microscopic features demonstrated cytoplasmic pattern of ligands and nuclear localization of GLI1. Tumour sections showed 54%, 65% and 75% positive staining against DHH, SHH, and GLI1 also mentioned in the Fig. 1. High intensity of protein expression was observed in patients with advanced pTNM stages and poorly differentiated nuclear grade. Hence a consistency of transcriptional profiling with protein profiling of SHH, DHH and GLI1 were also established. High expression of all pathway components especially GLI1 (HR = 1.79 (1.25–2.56), *p* < 0.05) demonstrated significant association with shorter DMFS (distant metastasis free survival) in grade III patients (Additional file 1: Figure S2). This information was coherent with expression profile of our dataset rendering hedgehog pathway as a marker of poor prognosis.

### Correlation of hedgehog pathway with metastasis and nodal involvement

Elevated expression of SHH and DHH correlated with N1+ nodal involvement in both datasets. Increase in mean copy numbers of SHH and DHH were associated with shorter overall survival in TCGA dataset. Similarly increase in mean copy number of SHH (R.E = 8.2 ± 3.7, *p* < 0.0001), DHH (R.E = 6.5 ± 4.4, *p* < 0.05) and GLI1 (R.E = 6.6 ± 3.9, *p* < 0.05) were associated with presence of lymph node involvement in Pakistani cohort. Similarly, patients having distant metastasis significantly demonstrated enhanced SHH, DHH and GLI1 expression with highest levels of DHH (R.E = 17.1 ± 2.2, *p* < 0.01) shown in the Fig. 2.

### Association of hedgehog pathway with ER, PR, Ki67 and HER2

In Pakistani cohort, all hedgehog molecules were strongly related to hormonal receptors (ER and PR) (r-value ranging from 0.71 to 0.87, p < 0.05). However, majority of hedgehog molecules did not show any significant correlation with HER2 as shown in Additional file 1: Table S2. A strong positive correlation was established between Ki-67 proliferation index and SHH, DHH, PTCH1 and GLI1 (r-value ranging from 0.60 to 0.78, p < 0.05) indicating their putative role in cell proliferation.

### Correlation of hedgehog pathway with molecular subtypes of breast cancer

**Fig. 3:**
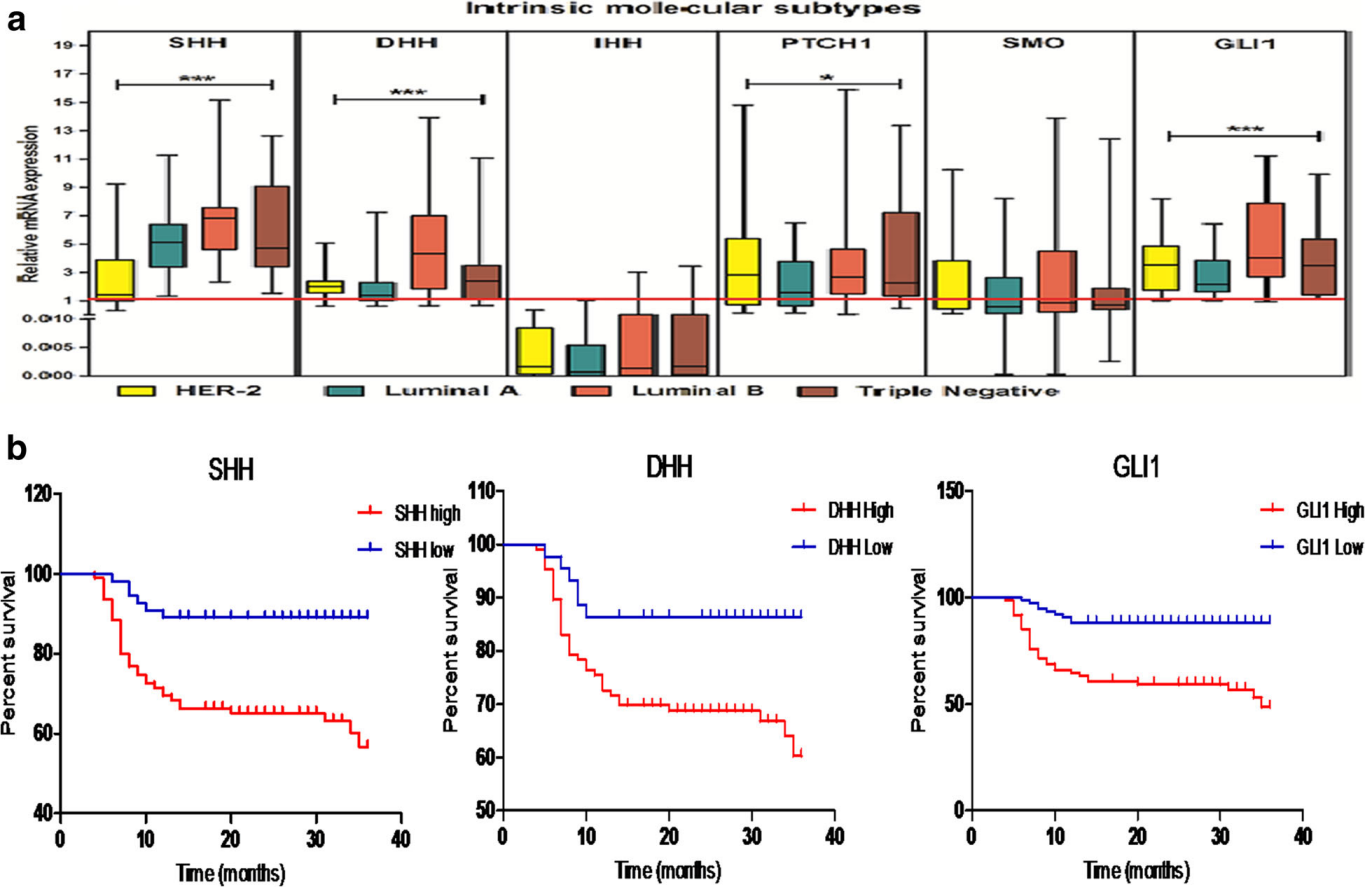
Correlation of Hedgehog molecules with Intrinsic Molecular Subtypes and Overall Survival of patients. **a**. Expression variation of hedgehog pathway in molecular subtypes (Luminal A, Luminal B, HER2 and Triple negative) of breast cancer patients evaluated using qPCR. (Kruskal-Wallis Anova, *p < 0.05, ***p < 0.0001). **b**. Kaplan Meier plots showing association of SHH, DHH and GLI1 with Overall survival in Pakistani population (red = high expression, blue = low expression, significant *p* < 0.05)

In TCGA dataset, triple negative subtype of patients showed significant over expression of DHH, PTCH1 and SMO. Following St. Gallen International Expert Consensus System, Pakistani cohort retains highest proportion of luminal-B (53%), followed by triple negative (18%), luminal-A(15%) and HER2 (14%) individuals. Both luminal B and triple negative subtypes representing patients showed significantly increase of SHH, DHH, PTCH1and GLI1 as shown in the Fig. 3. Furthermore, prognostic role of hedgehog pathway was assessed using the online available Kaplan Meier Plotter dataset (*n* = 626 patients, follow-up threshold = 15 yrs) [17]. Elevated expression of hedgehog pathway members was also associated with shorter distance metastasis free survival in patients diagnosed with luminal-B breast cancer patients as shown in the Additional file 1: Figure S3.

### High expression of SHH, DHH and GLI1 is poor predictor of overall survival

Due to prospective nature of study design, follow-up data for overall survival was collected for 36 to 40 months. Kaplan-Meier plots were generated using high and low expression of SHH, DHH and GLI1 (Fig. 3). Log-rank test was used to assess the effect of expression of SHH (HR = 2.97 (1.6–5.6), *p* = 0.0007), DHH (HR = 2.25 (1.2–4.4), *p* = 0.02) and GLI1 (HR = 4.06(2.2–7.5), *p* < 0.0001) which were all associated with poor predicted mortality. Based on univariate and multivariate Cox’s proportional regression analyses, elevated expression of SHH (HR = 3.94 (1.7–9.4), *p* = 0.002), DHH (HR = 2.72 (1.2–6.5), p = 0.02) and GLI1 (HR = 4.5 (2.2–9.4), *p* < 0.0001) were found to be independent predictor of overall survival. Furthermore patients having ER positive tumours (HR = 3.05 (1.36–6.89), *p* = 0.007) and distant metastasis (HR = 3.47 (1.2–9.7), p = 0.02) were found to be associated with shorter overall survival. Age (HR = 0.18 (0.07–0.44), *p* = 0.0003) was also found to be a significant predictor of mortality having association of older age with less hazard ratio and good prognosis (Table 2). Overall high expression of SHH, DHH and GLI1 was found to be an independent predictor of poor mortality in the present cohort.

**Table 2:** Univariate and multivariate Cox’s proportional hazard regression analyses of Pakistani breast cancer cohort for potential predictors of overall survival

Characteristics	Univariate analysis		Multivariate analysis	
	HR (95% CI)	*p* value	HR (95% CI)	*p* value
SHH			3.14 (1.3–7.7)	**0.01**
Age	0.18 (0.06–0.45)	**0.0003**	0.2 (0.08–0.56)	**0.002**
SHH	3.89 (1.6–9.2)	**0.002**		
ER	2.67 (1.2–6.0)	**0.02**	2.26 (0.97–5.25)	**0.05**
SHH	3.53 (1.5–8.4)	**0.004**		
PR	1.76 (0.9–3.5)	0.09	–	**–**
SHH	3.58 (1.5–8.6)	**0.004**		
HER-2	1.16 (0.6–2.1)	0.6	–	**–**
SHH	3.93 (1.7–9.3)	**0.002**		
Metastasis	2.96 (1.1–8.3)	**0.03**	2.62 (1.3–5.3)	**0.007**
SHH	3.79 (1.6–9.0)	**0.002**		
DHH			2.83 (1.2–6.8)	**0.02**
Age	0.16 (0.07–0.43)	**0.0002**	0.17 (0.1–0.5)	**0.0003**
DHH	2.94 (1.2–6.9)	**0.01**		
ER	2.78 (1.2–6.3)	**0.01**	2.00 (0.8–4.9)	0.13
DHH	2.42 (1.0–5.8)	**0.04**		
PR	1.97 (1.0–3.85)	**0.05**	2.04 (0.97–4.3)	**0.06**
DHH	2.56 (1.1–6.1)	**0.03**		
HER-2	1.16 (0.6–2.1)	0.62	–	**–**
DHH	2.72 (1.2–6.5)	**0.02**		
Metastasis	3.97 (1.9–7.9)	**0.0001**	2.9(1.4–5.9)	**0.003**
DHH	3.16 (1.3–7.5)	**0.009**		
GLI1			3.67 (1.7–7.9)	**0.0008**
Age	0.17 (0.07–0.43)	**0.0002**	0.18 (0.1–0.5)	**0.0004**
GLI1	4.67 (2.2–9.8)	**<0.0001**		
ER	2.43 (1.1–5.5)	**0.03**	2.45 (1.1–5.6)	**0.03**
GLI1	3.98 (1.9–8.4)	**0.0003**		
PR	1.26 (0.6–2.6)	0.52	–	**–**
GLI1	4.15 (1.9–9.1)	**0.0004**		
HER-2	1.01 (0.5–1.9)	0.98	–	**–**
GLI1	4.51 (2.2–9.5)	**<0.0001**		
Metastasis	2.54 (1.3–5.1)	**0.009**	1.85 (1.9–3.8)	**0.009**
GLI1	4.01 (1.9–8.5)	**0.0003**		

Bold values show significance at *p* < 0.05, covariates significant in univariate analyses were considered for multivariate analyses

### GANT61 induce apoptosis and restrict cell proliferation

**Fig. 4:**
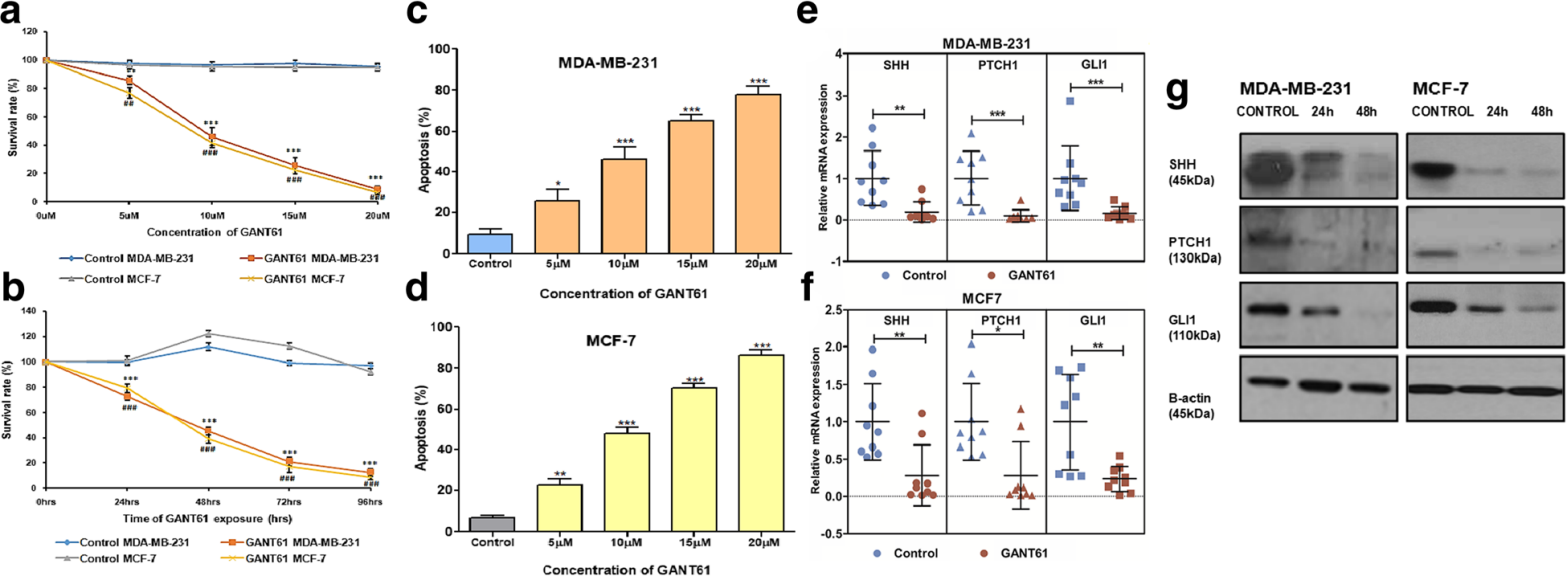
Effect of GANT61 on proliferation, apoptosis and hedgehog pathway. Inhibitory effect of GANT61 on proliferation and apoptosis of breast cancer cells was tested using in vitro models. Cell viability assays were conducted using CCK-8, to observe the effect of GANT61 on cell proliferation in MDA-MB-231 and MCF-7 in **a**. Dose dependent (5, 10, 15, 20 μM) and **b**. Time dependent manner (24, 48, 72, 96 h). Apoptosis was assessed using Annexin V at variable concentrations (5, 10, 15, 20 μM) of GANT61 in **c**.MDA-MB-231 and **d**.MCF-7 (Unpaired t test, *p < 0.05, **p < 0.001, ***p < 0.0001). IC50 of GANT61 was determined to be 10 μM. Inhibition of expression of hedgehog pathway after treatment with GANT61 (10 μM) for 48 h. Relative mRNA expression of SHH, PTCH1 and GLI1 using qPCR in **e**. MDA-MB-231, **f**. MCF-7 (Mann Whitney U test, *p < 0.05, **p < 0.001, ***p < 0.0001). **g**. Protein expression of SHH, PTCH1 and GLI1 using western blot having β-actin as internal control in both cell lines. All experiments were conducted in triplicates, performed thrice and values are represented as mean ± S.D.

Cell proliferation for both MDA-MB-231 and MCF-7 treated with variable concentrations (0, 5, 10, 15, 20 μM) of GANT61 were measured at 5 different time points respectively. GANT61 significantly inhibited proliferation of MDA-MB-231 and MCF-7 cells in a dose (Fig. 4a) and time-dependent (Fig. 4b) manner from their respective control groups (*p* < 0.0001). In order to determine apoptosis induction, Annexin V was used as apoptotic marker in FACS analysis. GANT61 induced substantial apoptosis in both cell lines in a concentration dependent fashion (Fig. 4c and d). Based on cell proliferation and apoptosis assay, 10 μM concentration of GANT61 showed ~50% reduction in cell viability. Impact of GANT61 in suppressing hedgehog pathway was assessed by measuring SHH, PTCH1 and GLI1 expressions using qPCR and western blot. GANT61 effectively inhibited GLI1 (R.E = 0.2 ± 0.1 in MDA-MB-231 and R.E = 0.2 ± 0.17 in MCF-7, *p* < 0.05). Being targets of GLI1, expression of SHH (R.E = 0.18 ± 0.2 in MDA-MB-231 and R.E = 0.3 ± 0.4 in MCF-7, *p* < 0.05) and PTCH1 (R.E = 0.09 ± 0.14 in MDA-MB-231 and R.E = 0.3 ± 0.4 in MCF-7, *p* < 0.05) were also repressed after treatment with GANT61 as shown in the Fig. 4.

### GANT61 reduced metastatic potential by decreasing cell migration and invasion

**Fig. 5:**
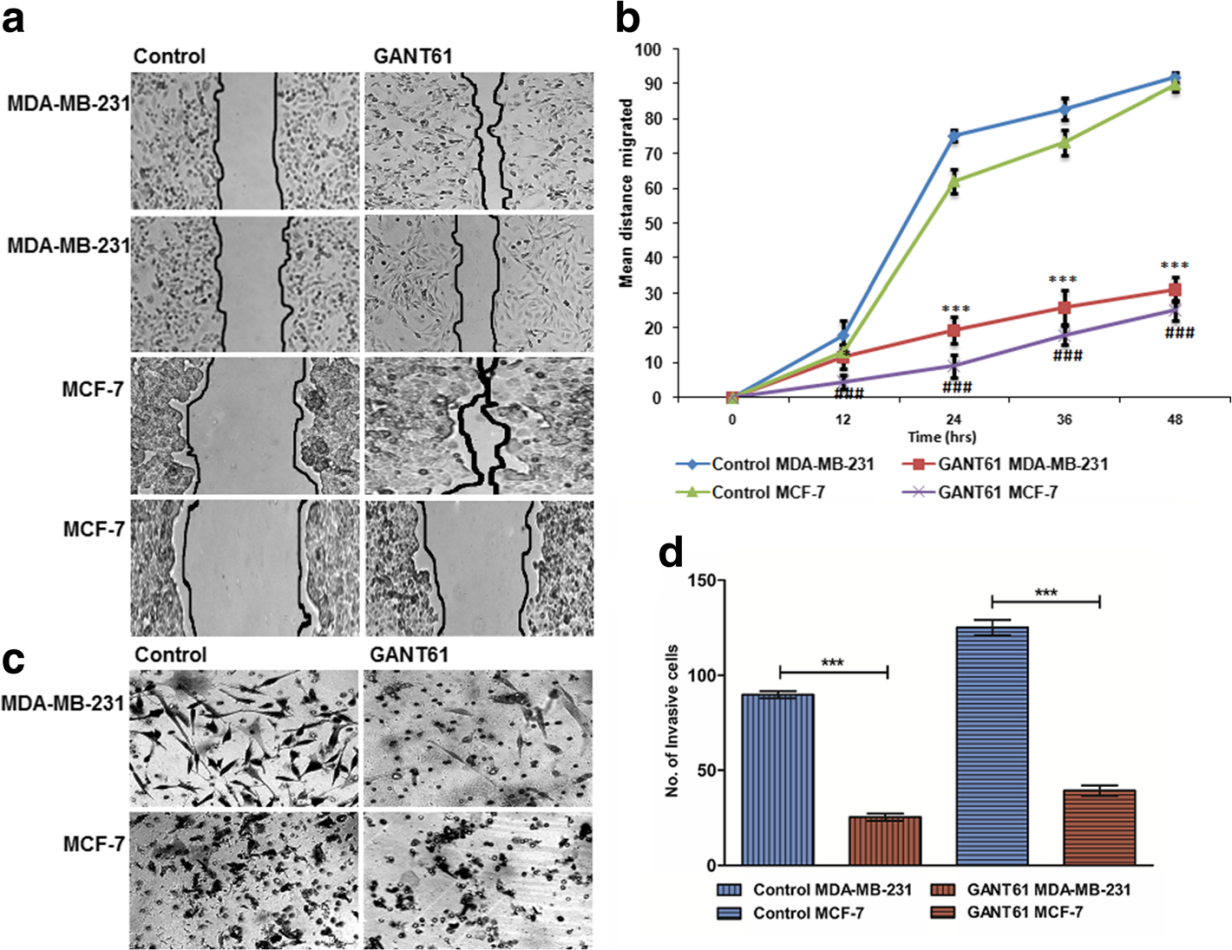
Effect of GANT61 on motility and invasion of breast cancer cells. MDA-MB-231 and MCF-7 were treated with the conditional medium containing 10 μM GANT61 and the control medium. **a**. Migration was assessed using scratch assay and readings were taken after every twelve hour for 48 h, upper panel of both cell lines represent 0 h and the lower panel represents 48 h. **b**. Graph showing difference of distance migrated between untreated and treated cells at each time point of scratch assay. **c**. Invasion was assessed using Boyden chamber transwell assays and difference between treated and untreated cells was evaluated after 24 h. **d**. Graphical representation of number of cells invaded as mean ± S.D. between untreated and treated cells in both cell lines. (Unpaired t test, ***, ###*p* < 0.0001). All experiments were conducted in triplicates and were performed thrice

Furthermore, effect of hedgehog signaling on motility of breast cancer cells using an in vitro wound-healing assay. MDA-MB-231 and MCF-7 cells were treated with pre-conditional medium containing GANT61 (10 μM) and control medium containing DMSO. GANT61 inhibited cell migration in both cell lines in contrast to their respective control cell lines (*p* < 0.0001) as shown in the Fig. 5. Moreover, the effect of hedgehog signaling on the invasive ability of breast cancer cells was also evaluated using matrigel invasion assay. The ability of breast cancer cells to invade matrigel was markedly reduced in cells that were treated with GANT61 (*p* < 0.0001), suggesting that hedgehog signaling has an essential role in the migration and invasiveness of breast cancer cells.

## Discussion

Expression pattern of all aforementioned genes of hedgehog pathway are in line with earlier studies in different cancers [18–21]. Interestingly, lack of IHH association with clinical parameters is also observed in breast and prostate cancers [22, 23] in line with the given cohort. However, up regulation of IHH observed among Chinese breast cancer cohort is contradictory to our findings [24]. This may be attributed to population specific genotypic variations, multiple hedgehog ligands targeting PTCH1 and sensitivity of detection technique.

Over-expression of SHH, DHH and GLI1 was frequently observed in triple negative and luminal-B subtypes of the given cohort. In triple negative patients, activated hedgehog pathway was significantly related with advanced tumour stages and poor prognosis as has already been reported [25, 26]. Hence suppression of hedgehog pathway may provide a novel therapeutic strategy for treating TNBC. On the other hand, association of hedgehog pathway with luminal subtypes can also be explained based on positive correlation of different hedgehog molecules with ER in the cohort. Co-expression of ER and SHH was present among 60% of the cohort patient’s, indicating molecular crosstalk as mentioned previously [27]. Similarly, strong positive correlation between hedgehog members (DHH, SHH, GLI1) and high Ki-67indexwith values 75, 80 and 85% respectively was observed. These findings corroborate interplay of hedgehog pathway in tumour growth of luminal B subtype patients. Earlier it has been shown that inhibition of GLI1 expression via RNAi mediated depletion restricted cell growth in ER positive tamoxifen resistant cells [28]. Furthermore, role of hedgehog pathway towards tumourigenesis in endocrine therapy resistant patients has also been established [29]. Based on these findings, inclusion of hedgehog pathway as novel therapeutic target in treating patients belonging to both TNBC and luminal-B subtypes is strongly recommended.

Generally, aggressive molecular subtypes noted among young women are associated with worse outcome [30]. Hence, identification of alternate predictive and therapeutic biomarker is a necessity for these young patients. In the given study, alteration of hedgehog pathway was frequently observed among young Pakistani patients. Keeping in view increasing incidence of breast cancer in Pakistani women, devising novel therapeutic strategies are an absolute requirement. In the given cohort, patients < 45 yrs. showed frequent over expression of SHH, DHH and GLI1 contributing towards aggressive tumourigenesis. Interestingly earlier findings also indicate that pre-menopausal patients showed more aggressive tumour progression in comparison to post menopausal population [31]. Hence elevated expression of SHH, DHH and GLI1 was also observed in pre-menopausal patients signifying their association with poor prognosis. Thus it is necessary to adapt a molecular screening driven approach to refine treatment for younger breast cancer patients including hedgehog inhibitors in adjuvant therapy regimen.

Over-expression of SHH, DHH and GLI1 was significantly correlated with advanced stages and tumour grades of the cohort. Activation of hedgehog pathway in advance cancer stages has been reported previously in breast [25, 32], ovarian [18], renal [19] and prostate cancer [10]. Concomitantly, dysregulation of SHH, DHH and GLI-1 in the cohort has also been related with nodal involvement and metastasis. In another study, significant relevance of hedgehog ligands with metastasis and breast cancer specific death has been reported [33]. Similar results were obtained at both mRNA and protein levels in Taiwanese and German populations [6, 34]. These findings strongly suggest role of hedgehog pathway in breast cancer progression and metastatic spread.

High expression of SHH, DHH and GLI1 was also found to be an independent predictor of overall survival in this cohort along with age, distant metastasis and ER expression. However, SMO has a limited clinical relevance as observed in this cohort. No significant association of SMO with patient’s age, metastasis or other relevant parameters has been established in this study. Although, numerous SMO inhibitors like GDC-0449 (Vismodegib) have entered clinical trials for triple negative breast cancer patients but their efficacy still remains uncertain. Benvenuto et al. have recently proven that targeting hedgehog pathway using antagonists like GANT61 that act downstream of SMO is a more efficient strategy than using antagonists against SMO in breast cancer [35]. Furthermore non-canonical transcriptional activation of hedgehog pathway has also been extensively documented [36]. Hence, role of GANT61 as potential inhibitor of GLI1 against metastatic cascade has been deciphered. In this study, IC50 determined for both MCF-7 (ER + ve) and MDA-MB-231 (ER-ve) cell lines, was 10 μM. It showed maximum reduction of cell proliferation after 48 h. This concentration of GANT61 was enough to suppress expression of GLI1 and transcriptional activation of PTCH1 and SHH as mentioned earlier [35]. Furthermore, GANT61 significantly reduced both motility and invasion thus efficiently demonstrating its potential to restrict cancer metastasis.

## Conclusion

Expression analysis of hedgehog pathway at early stage is highly recommended especially for young patients. Furthermore, this is the first ever report regarding evaluation of GANT61 potential role on metastasis suppression. Clinical and in-vitro functional assays findings strongly emphasize on hedgehog pathway’s putative role as potential cancer therapeutic target in breast cancer patients.

## Additional file

Additional file 1: **Figure S1.** Expression profile of hedgehog pathway genes in Oncomine database. **Figure. S2.** Kaplain Meier plot showing correlation of high expression of GLI1 with shorter distance metastasis free survival (DMSF) in grade 3 patients (*N* = 458, 15 years follow-up) in the KM plotter database. **Figure. S3.** Kaplan Meier plots showing association of SHH, DHH, IHH, PTCH1, SMO and GLI1 with Distant Metastasis Free Survival (DMSF) in patients belonging to luminal B subtype (*N* = 156, 15 years follow-up) (red = high expression, black = low expression, significant *p* < 0.05). **Table S1.** Primer sequences for qRT-PCR. **Table S2.** Correlation among hedgehog pathway molecules and their association with Ki-67, ER, PR and HER-2. (DOCX 742 kb)

## Abbreviations

BCA: Bicinchoninic Acid
DHH: Desert hedgehog
DMSO: Dimethyl sulfoxide
ER: Estrogen receptor
GLI1: glioma-associated oncogene homolog 1
HER-2: Human Epidermal growth factor Receptor 2
IHH: Indian hedgehog
IRS: Immuno-Reactive-Scores
Ki-67: Proliferative marker
PR: Progesterone receptor
PTCH1: Patched1
SHH: Sonic hedgehog
SMO: Smoothened
