# Supplementary material for: Involvement of hedgehog pathway in early onset, aggressive molecular subtypes and metastatic potential of breast cancer
Source: Cell Commun Signal. 2018 Jan 8;16:3. doi: 10.1186/s12964-017-0213-y (PMC5795292; doi:10.1186/s12964-017-0213-y)
Supplement: Additional file 1: — Figure S1. Expression profile of hedgehog pathway genes in Oncomine database. Figure. S2. Kaplain Meier plot showing correlation of high expression of GLI1 with shorter distance metastasis free survival (DMSF) in grade 3 patients (N = 458, 15 years follow-up) in the KM plotter database. Figure. S3. Kaplan Meier plots showing association of SHH, DHH, IHH, PTCH1, SMO and GLI1 with Distant Metastasis Free Survival (DMSF) in patients belonging to luminal B subtype (N = 156, 15 years follow-up) (red = high expression, black = low expression, significant p < 0.05). Table S1. Primer sequences for qRT-PCR. Table S2. Correlation among hedgehog pathway molecules and their association with Ki-67, ER, PR and HER-2. (DOCX 742 kb) [file 12964_2017_213_MOESM1_ESM.docx]

**Additional file**

Figure S1:

**
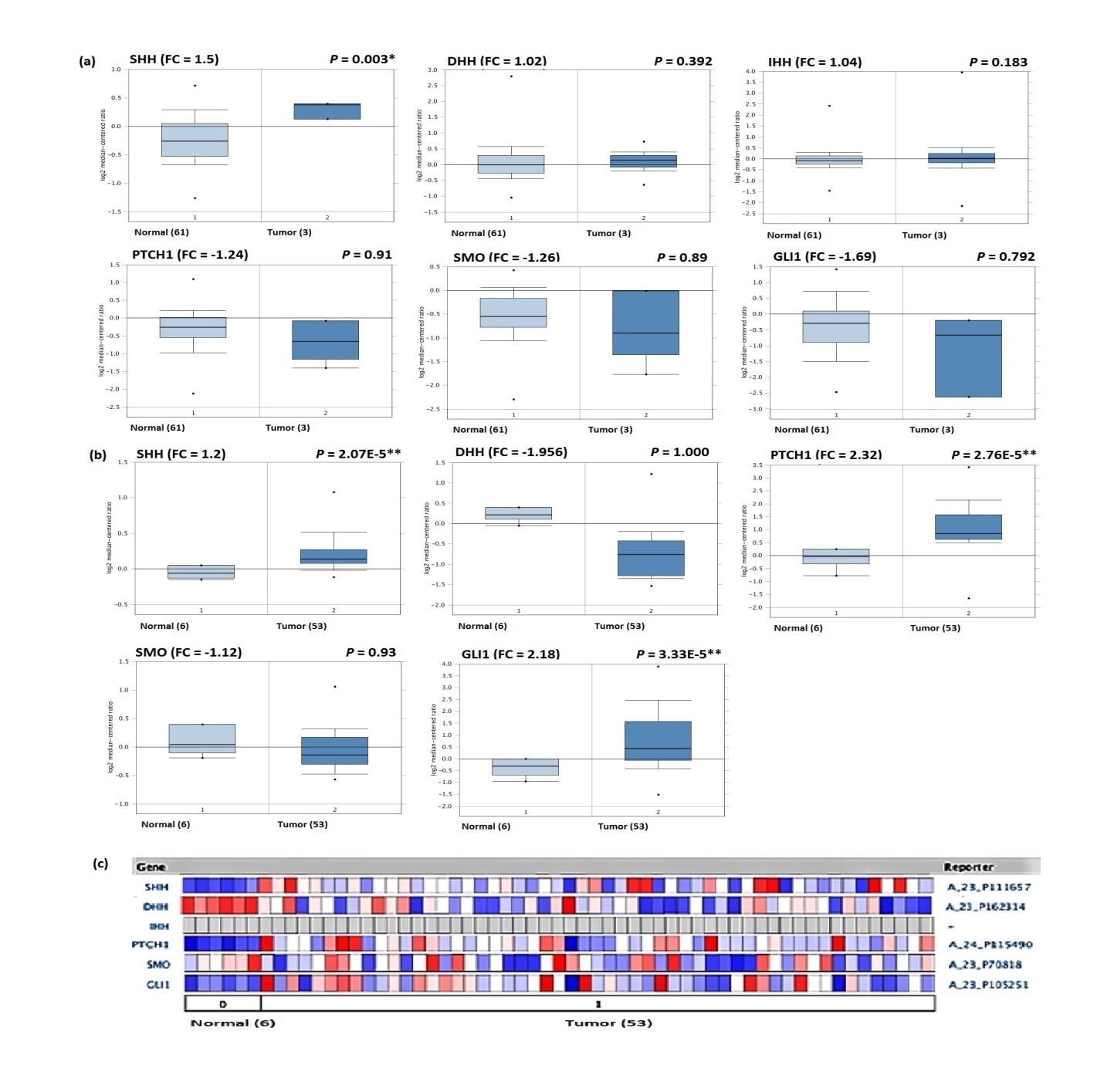
**

Expression profile of hedgehog pathway genes in Oncomine database. a). Box whisker graphs showing log-median centered ratio of hedgehog pathway genes in TCGA breast. b). Box whisker graphs showing log-median centered ratio of hedgehog pathway genes in Finak breast. c). Heatmap of Finak breast showing expression pattern of hedgehog pathway genes.

Figure S2:


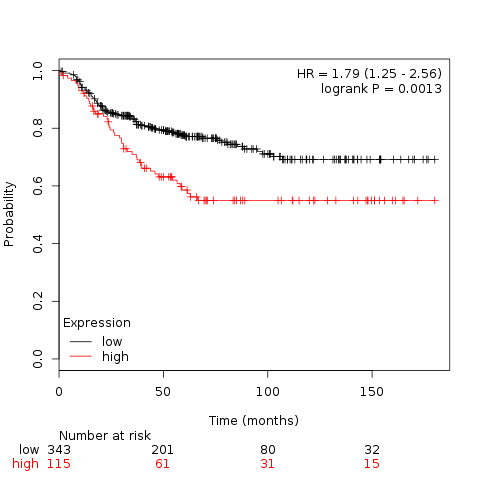


Kaplain Meier plot showing correlation of high expression of GLI1 with shorter distance metastasis free survival (DMSF) in grade 3 patients (N=458, 15 years follow-up) in the KM plotter database.

Suppl. Figure 3:


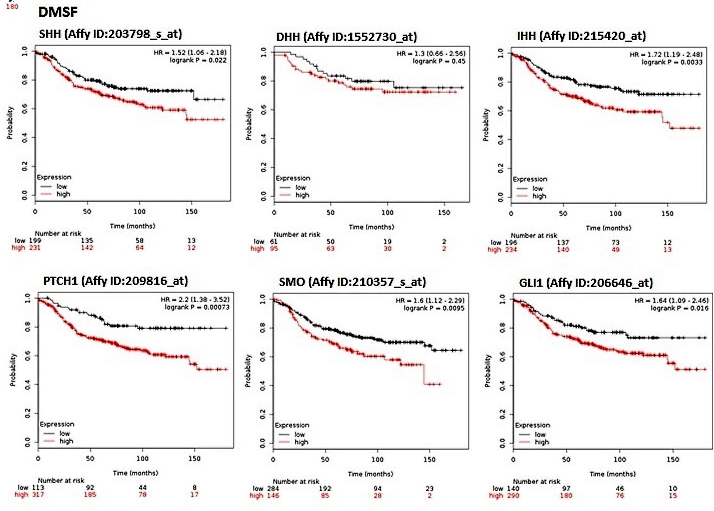


Kaplan Meier plots showing association of SHH, DHH, IHH, PTCH1, SMO and GLI1 with Distant Metastasis Free Survival (DMSF) in patients belonging to luminal B subtype (N=156, 15 years follow-up) (red = high expression, black = low expression, significant p<0.05).

Table S1: Primer sequences for qRT-PCR.

| Primer name | Forward primer | Reverse primer | Product length |
| --- | --- | --- | --- |
| SHH | CTTCCTCACTTTCCTGGACCG | GGTGGCCGAGTCGTTGT | 136 |
| IHH | CTCCTCTGCCATGAAGATACAC | CTCAAGGTCTCTAGGAGAGAGG | 141 |
| DHH | GCCTGCTGGCTGAGATATG | CGGTATCACCTCCTCTCAGTA | 132 |
| PTCH | CCTCGGGAAACCAGAGAATATG | AAACTCCTGTGTAGGTCGTAAAG | 134 |
| SMO | CTGCCACTTCTACGACTTCTTC | CTTGATCTCACAGTCAGGGATG | 127 |
| GLI | CACATCCACAGCCTCTCTTT | CCTGGGTTCTGAAGGAAGATAAT | 110 |
| Ki-67 | GCCTTGGTCTCTTGGGAATAC | GGAGATTAGGAGCCAGTTTGAG | 123 |
| ER | CCACCAACCAGTGCACCATT | GGTCTTTTCGTATCCCACCTTTC | 108 |
| PR | ATT ACC AGT GTT CCC GTC TTC | CCT GTA CTT CCT CCA GCA TAA | 111 |
| HER-2 | TTGAGTCCATGCCCAATCC | GTGTTCCATCCTCTGCTGTC | 150 |
| β-actin | ATGATATCGCCGCGCTCA | CGCTCGGTGAGGATCTTCA | 150 |

Table S2: Correlation among hedgehog pathway molecules and their association with Ki-67, ER, PR and HER-2*

|  | SHH | DHH | IHH | PTCH | SMO | GLI-1 | Ki-67 | HER-2 | ER | PR |
| --- | --- | --- | --- | --- | --- | --- | --- | --- | --- | --- |
| SHH | | **0.79** | **0.80** | **0.79** | **0.83** | **0.60** | **0.65** | 0.11 | **0.77** | **0.72** |
| DHH |  |  | **0.77** | **0.76** | **0.81** | **0.65** | **0.63** | 0.10 | **0.78** | **0.72** |
| IHH |  |  |  | **0.85** | **0.87** | **0.49** | **0.48** | 0.12 | **0.87** | **0.80** |
| PTCH |  |  |  |  | **0.83** | **0.49** | **0.60** | 0.12 | **0.81** | **0.74** |
| SMO |  |  |  |  |  | **0.52** | **0.23** | **0.20** | **0.85** | **0.81** |
| GLI-1 |  |  |  |  |  |  | **0.78** | 0.16 | **0.71** | **0.50** |
| Ki-67 |  |  |  |  |  |  |  | 0.14 | **0.77** | **0.71** |
| HER-2 |  |  |  |  |  |  |  |  | 0.13 | 0.16 |
| ER |  |  |  |  |  |  |  |  |  | **0.87** |
| PR |  |  |  |  |  |  |  |  |  |  |

***Spearmen correlation, all bold values are significant having p<0.05**
